# Dietary Fat Sources Affect Hepatic Health, Performance and Gut Microbiota Composition in Laying Hens as Model

**DOI:** 10.1002/vms3.70665

**Published:** 2025-10-21

**Authors:** Humera Hamid, Yao Jun Liu, Wen Xiang Li, Shi Meng Huang, Li Hong Zhao, Lan Jiao Xu, Ming Ren Qu, Qiu Gang Ma

**Affiliations:** ^1^ Jiangxi Province Key Laboratory of Animal Nutrition and Feed Jiangxi Agricultural University Nanchang China; ^2^ State Key Laboratory of Animal Nutrition & Feeding College of Animal Science and Technology China Agricultural University Beijing China; ^3^ Feed Safety and Healthy Livestock Beijing Jingwa Agricultural Innovation Center Beijing China

**Keywords:** dietary fat, fatty liver disease, gut microbiota, laying hens, margarine, milk cream, poultry nutrition

## Abstract

Non‐alcoholic fatty liver disease (NAFLD) is a growing concern in both human and animal health, with nutritional strategies playing a key role in its management. This study used a laying hen model to evaluate the effects of two high‐fat diets, one containing margarine (MAR) and the other natural milk cream (NC) on hepatic health, egg production and gut microbiota composition. Both diets were formulated with 8% fat but differed in fatty acid profiles: MAR was rich in lauric and stearic acids, whereas NC contained more palmitic and oleic acids. Compared to NC, MAR‐fed hens showed significantly higher total cholesterol, low‐density lipoprotein cholesterol (LDL‐cholesterol), liver fat, relative liver weight, abdominal fat and serum markers of liver damage (aspartate aminotransferase [AST], alkaline phosphatase [ALP], gamma‐glutamyl transferase [GGT] and adiponectin) (*p* < 0.05). Microbiota analysis revealed that although Firmicutes and Bacteroidetes dominated in both groups, MAR‐fed hens had lower microbial diversity (Shannon index) and altered relative abundance of Verrucomicrobia, Peptostreptococcaceae and Turicibacter (*p* < 0.05), indicating microbial dysbiosis. These findings demonstrate that the type of dietary fat—independent of total fat content—strongly influences liver function and gut microbial balance in poultry. The novelty of this study lies in showing how different fat sources, despite equal inclusion levels, can distinctly modulate the gut–liver axis. This provides practical feed formulation insights and advances understanding of the gut–liver axis in animal health.

## Graphical abstract

This study shows that margarine and natural cream, despite equal fat levels, had distinct effects on laying hens. Margarine altered blood lipid markers and reduced gut microbial diversity, whereas natural cream maintained a more balanced microbiota, emphasizing the role of fat source in modulating the gut–liver axis.

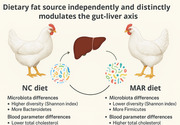

## 1 Introduction

Non‐alcoholic fatty liver disease (NAFLD) is a hepatic disorder characterized by excessive fat accumulation in the liver, exhibiting a range of severity from mild hepatic steatosis to severe non‐alcoholic steatohepatitis (NASH), progressing further to cirrhosis and hepatocellular carcinoma (HCC) (Cotter et al. 2020). The mechanisms involved, particularly the contribution of nutritional and metabolic factors influencing NAFLD pathogenesis, are complex and not yet fully understood (Ullah et al. 2019). Many studies have confirmed that laying hens serve as a suitable model for studying NAFLD and liver fat accumulation relevant to humans (Tsai et al. 2017; Hamid et al. 2019). Laying hens are a valuable model due to their high metabolic demand for egg production and physiological similarities with human lipid metabolism (Trott et al. 2014).

Margarine (MAR) and natural milk cream (NC) were selected due to their contrasting fatty acid profiles, providing a controlled model to investigate the effects of saturated fatty acid types on liver health and gut microbiota, despite their uncommon use in commercial poultry diets. This MAR is special in that it contains no trans fats—unlike most MARs that do—yet is rich in stearic acid and lauric acid, two saturated fats with distinct physiological effects. Stearic acid has been reported to have a neutral effect on cholesterol levels, whereas lauric acid is recognized for its antimicrobial activity but also has implications for lipid metabolism. In contrast, milk cream contains higher levels of palmitic acid, a saturated fat associated with increased lipid accumulation in the liver (Murru et al. 2022), and oleic acid, a monounsaturated fatty acid known to reduce inflammation and improve lipid profiles (Zeng et al. 2020; Castillo et al. 2024). Although palmitic acid has lipotoxic properties that contribute to NAFLD by increasing hepatic fat deposition and insulin resistance (Murru et al. 2022), the presence of oleic acid—a monounsaturated fat—may explain why hens fed the NC diet exhibited reduced liver fat accumulation compared to those on the MAR diet. These variations in fatty acid composition provide a unique opportunity to explore how different dietary fat sources influence the progression of fatty liver disease and the composition of the caecal microbiota in laying hens. As the gut microbiota plays a crucial role in liver function and overall metabolic regulation, it is important to understand how specific fats may affect both the gut ecosystem and hepatic health. Dysbiosis, or microbial imbalance, has been closely linked with NAFLD progression, liver inflammation and steatosis, suggesting a bidirectional gut–liver axis. The aim of this study was to investigate the impact of different dietary fats on fatty liver development and gut microbiota composition using laying hens as a model. The novelty of this work lies in directly comparing two fat sources with contrasting fatty acid profiles—MAR (rich in lauric and stearic acids) and NC (rich in palmitic and oleic acids)—while keeping the total fat inclusion level constant. This design allows us to isolate the effects of fat type, independent of fat quantity, on liver function and gut microbial balance. A limitation of this study is the absence of egg quality data, and egg quality traits such as shell strength, yolk composition and albumen quality are critical parameters for evaluating the overall nutritional and commercial relevance of dietary interventions in poultry production, which should be addressed in future research to strengthen applicability in poultry production. Nevertheless, the present findings provide insight into fat–microbiota–liver interactions and may guide the development of dietary strategies to prevent or mitigate fatty liver disease in poultry and potentially in humans.

## 2 Materials and Methods

### 2.1 Birds, Diets and Housing

All experimental procedures were approved by the Institutional Animal Care and Use Committee of University (Approval No. AW80011202‐1‐1) and adhered to national and international guidelines for the ethical use of animals in research. A total of 40 clinically healthy, 52‐week‐old *Hy‐Line White* laying hens were randomly assigned to two dietary treatment groups (*n* = 20 per group). Each bird was housed individually in stainless steel ladder‐type cages (45 × 45 × 45 cm^3^) equipped with separate feeders and nipple drinkers to allow precise feed intake monitoring. Daily feed intake was measured individually. Feed refusals and spillage were collected and subtracted from the total feed offered to calculate accurate intake per bird. Feed and water were provided ad libitum throughout the trial. The poultry house was maintained at 26°C ± 2°C with a relative humidity of 50% ± 10%. A photoperiod of 16 h light and 8 h dark was applied to simulate standard laying conditions. The feeding trial was conducted over a period of 10 weeks. Experimental diets were formulated on the basis of NRC (1994) recommendations and the *Hy‐Line White* Management Manual. Detailed ingredient compositions are shown in Table 1a. Two high‐energy diets were prepared: one containing NC and the other containing MAR as the primary fat sources. Both fat sources contributed approximately 8% added fat, formulated to increase dietary metabolizable energy by 15% compared to a standard layer ration. The NC diet was enriched with palmitic and oleic acids, whereas the MAR diet contained higher levels of lauric and stearic acids. Estimated apparent metabolizable energy (AME) was approximately 3464 kcal/kg for the MAR diet and 3352 kcal/kg for the NC diet, based on digestibility data. These differences were accounted for during data interpretation. Both fats were stored under refrigeration in sealed containers to maintain oxidative stability. Fresh feed was prepared weekly to ensure uniform fat distribution and minimize rancidity, and careful homogenization was performed to ensure uniform fat distribution and consistency across batches. Actual fat content of diets after mixing was verified using Soxhlet extraction, confirming that formulated and analysed values were in agreement.

**TABLE 1a:** Composition and nutrient level of different diets (as‐fed basis).

Ingredients	NC	MARG
Corn	44	44
Corn starch	12	12
Soybean meal dehulled	25	25
Natural cream	8	—
Trans fat(margarine)	—	8
Limestone	8.3	8.3
DHCP	1.5	1.5
Salt	0.3	0.3
Vitamins premix^b^	0.03	0.03
Micro‐minerals premix^c^	0.3	0.3
dl‐Methionine	0.17	0.17
Zeolite	0.4	0.4
	100	100
AME	3.09	3.09
CP^a^	15.2	15.2
Lys	0.82	0.82
Met	0.4	0.4
Met + Cys	0.64	0.64
Calcium^a^	3.56	3.56
Phosphorus	0.54	0.54
Available phosphorus	0.35	0.35

*Note*: Formulated according to NRC and Hy‐Line Manual recommendations, with 8% natural milk cream (NC) or margarine (MAR) inclusion.

Abbreviations: AME, apparent metabolizable energy; MAR, margarine; NC, natural milk cream.

^a^ Crude protein and calcium levels were analysed. Each value was based on triplicate determinations. However, all other nutrient levels were calculated.

^b^ Provided per kilogram of diet: 8500 IU of vitamin A (retinol acetate), 3600 IU of vitamin D3, 21 IU of vitamin E (dl‐α‐tocopherol acetate), 4.2 mg of vitamin K3 (menadione 7 dimethpyrimidinol), 3.0 mg of vitamin B1 (thiamin mononitrate), 10.2 mg of vitamin B2 (riboflavin), 45 mg of niacin, 15 mg of calcium pantothenate, 5.4 mg of vitamin B6 7 (pyridoxine), 0.15 mg of biotin, 0.9 mg of folic acid and 0.024 mg of vitamin B12.

^c^ Provided per kilogram of diet: 60 mg of Fe (FeSO_4_·7H_2_O), 80 mg of Zn (ZnSO_4_·7H_2_O), 60 mg Of Mn (MnSO_4_·H_2_O), 8 mg Cu (CuSO_4_·5H_2_O), 0.35 mg of I (KI) and 0.3 mg of Se (Na_2_SeO_3_).

The fatty acid profiles of the experimental diets were analysed using gas chromatography (Table 1b). Individual body weights were recorded at the start of the experiment and every 2 weeks following an overnight fast. Hen‐day egg production and egg weight were recorded daily.

**TABLE 1b:** Fatty acid composition (%) of the fat sources used in experimental diets: natural milk cream and margarine.

Fatty acid	NC (% of total FA)	MAR (% of total FA)
C6:0	1.70	0.23
C8:0	1.36	3.51
C10:0	3.66	3.13
C12:0	7.01	37.98
C13:0	0.13	0.04
C14:0	16.16	11.35
C14:1	1.15	—
C15:0	1.54	0.01
C16:0	37.83	7.51
C16:1	1.74	—
C17:0	1.00	0.03
C18:0	13.92	21.40
C18:1*n*9c	27.27	1.08
C18:2*n*6c	2.02	0.05
C18:3*n*3	0.79	—
C20:0	0.19	0.26
C22:0	0.19	0.05
C23:0	0.10	0.02
C24:0	0.15	0.07
**Total**	**100**	**100**

*Note*: Fatty acid composition (% of total identified fatty acids) of diets. Values are expressed as percentage of total identified fatty acids measured by GC.

Abbreviations: MAR, margarine; NC, natural milk cream.

### 2.2 Sampling Procedures and Tissue Collection

At the end of the feeding period (62 weeks of age), following an overnight (8 h) feed withdrawal, nine hens per group were selected for detailed sampling. Birds were selected randomly using a computer‐generated randomization scheme to represent the average body weight of each group, ensuring that sampling was unbiased and representative. Birds were euthanized by exsanguination through severing the jugular vein, in accordance with institutional humane slaughter protocols to minimize distress and ensure rapid death. Blood was collected immediately post‐slaughter into anticoagulant‐free tubes, allowed to clot at 4°C and centrifuged at 3000 × *g* for 15 min to separate serum, which was aliquoted and stored at −80°C until biochemical analyses. Each liver was excised, weighed and macroscopically assessed for fatty infiltration using a semi‐quantitative scoring system modified from Polin and Wolford (1977), which has been widely applied in avian fatty liver studies. The scoring scale ranged from 1 to 4, defined as: FS1—absence of visible lipid; FS2—mild lipid accumulation; FS3—moderate lipid deposition with visible pallor; FS4—severe lipid infiltration with apparent fibrosis. Representative liver tissue samples were fixed in 10% neutral‐buffered formalin for histopathological examination. Total hepatic lipid content was also determined gravimetrically. Caecal digesta were collected aseptically from each bird, snap‐frozen in liquid nitrogen and stored at −80°C for subsequent microbiota profiling.

### 2.3 Quantification of Hepatic Lipid Content

Hepatic lipids were extracted using the Folch method (Folch et al. 1957), with minor modifications to optimize extraction for poultry liver tissue. Total lipid content of liver tissue was quantified by ether extraction using an ANKOM XT15 Automated Extractor (ANKOM Technology, Macedon, NY, USA). Approximately 1 g of minced liver was weighed into specialized filter bags, oven‐dried at 102°C for 3 h, cooled in a desiccator, reweighed and subjected to solvent extraction at 60°C for 60 min. Extracted samples were oven‐dried again at 105°C for 30 min, cooled and weighed to determine fat loss by gravimetric difference, calculated as

$$\mathrm{Fat}\left(\%\right)=\left(\mathrm{WBD}-\mathrm{WAX}\right)\;\times \;100\;/\;W\mathrm{sample}$$

where WBD is the weight before drying; WAX is the weight after extraction; *W* sample is the initial tissue weight.

### 2.4 Histopathological Evaluation

Liver samples were processed for both paraffin embedding and cryosectioning. Paraffin‐embedded sections (5 µm) were stained with haematoxylin and eosin (H&E) to assess general hepatic architecture and with Picrosirius red to visualize collagen fibres and grade fibrosis, following Lattouf et al. (2014). For neutral lipid visualization, fresh liver samples were embedded in optimal cutting temperature (OCT) compound, cryosectioned at −20°C and stained with Oil Red O. All slides were examined independently by a board‐certified hematopathologist blinded to the treatment groups. Liver lesions were scored semi‐quantitatively based on the NASH Clinical Research Network Scoring System and the FLIP Consortium criteria (Goodman et al. 2007). The following parameters were scored: macrovesicular steatosis (0–3), lobular inflammation (0–3), hepatocyte ballooning (0–2) and fibrosis stage (0–4).

### 2.5 Serum Biochemical Assays

Serum concentrations of total cholesterol, triglycerides, high‐density lipoprotein cholesterol (HDL‐C), low‐density lipoprotein cholesterol (LDL‐C), uric acid and glucose were quantified using validated enzymatic colorimetric kits (Sentinel Diagnostics, Milan, Italy). Liver enzyme activities (alanine aminotransferase, aspartate aminotransferase [AST], alkaline phosphatase [ALP], creatinine and gamma‐glutamyl transferase [GGT]) were determined with an Advia 1800 Clinical Chemistry Analyser (Siemens Healthcare Diagnostics, Germany). Serum adiponectin and cytokine levels (IL‐4, IL‐6 and IL‐10) were measured using species‐specific ELISA kits (Hölzel Diagnostika, Germany; FineTest, Wuhan, China). The ratio of serum creatinine to body weight was calculated as an additional indicator of metabolic status.

### 2.6 Microbiota DNA Extraction and Sequencing

Genomic DNA was extracted from caecal contents using the QIAamp Fast DNA Stool Mini Kit (Qiagen), following Dewar et al. (2013) with slight modifications for avian gut samples. DNA purity and concentration were verified by NanoDrop spectrophotometry and agarose gel electrophoresis. The V3–V4 hypervariable region of the bacterial 16S rRNA gene was amplified using primers 338F and 806R. PCR products were purified, quantified and sequenced on an Illumina MiSeq PE300 platform (Illumina, San Diego, USA). Raw sequencing reads were quality‐filtered with Trimmomatic and assembled with FLASH. OTUs were clustered at 97% sequence identity using QIIME against the SILVA reference database. Alpha diversity indices (Shannon, Simpson) were calculated with MOTHUR. Beta diversity (Bray–Curtis dissimilarity) and principal coordinates analysis were conducted in R (Vegan and Pheatmap packages). Partial least squares discriminant analysis (PLS‐DA) was applied for multivariate visualization. Raw sequence data are available from the corresponding author upon reasonable request.

### 2.7 Statistical Analysis

Data were analysed using SPSS version 20.0 (SPSS Inc., Chicago, IL, USA). Assumptions of normality and homogeneity of variances were verified by Shapiro–Wilk and Levene's tests, respectively. Potential outliers were screened using boxplots and Grubbs’ test; no outliers were removed arbitrarily. Between‐group comparisons for zootechnical parameters, liver metrics, serum and biochemical indices were performed using independent samples *t*‐tests. Because histological scores are ordinal variables, they were analysed using the Kruskal–Wallis test, followed by pairwise Mann–Whitney *U*‐tests when appropriate. For categorical distributions (e.g., fibrosis stage categories), Fisher's exact test was applied. For microbial diversity indices and relative abundances, non‐parametric tests were used where appropriate. Results are reported as mean ± standard deviation (SD). Statistical significance was set at *p* < 0.05.

## 3 Results

### 3.1 Body and Organ Weights

After 10 weeks of feeding high‐fat diets, body weight did not differ significantly between hens fed NC or MAR. The liver weight, total liver fat and abdominal fat pad of hens fed the MAR diets were significantly higher than those from NC group (*p* < 0.05, Table 2).

**TABLE 2:** Body weight, relative liver weight and abdominal fat percentage in laying hens after 10 weeks on NC and margarine (MAR) diets.

Parameters	NC group	MARG group	*p* value
Body weight	1.48 ± 0.07	1.46 ± 0.04	0.26
Relative liver weight	1.97^b^ ± 0.20	2.33^a^ ± 0.56	0.01
Relative heart weight	0.35 ± 0.03	0.36 ± 0.001	0.15
Relative fat weight	2.05^b^ ± 0.20	3.28^a^ ± 0.94	0.05
liver fat weight	4.32^b^ ± 0.16	7.71^a^ ± 0.35	0.05

*Note*: Means bearing different superscript letters differ significantly *p* < 0.05.

### 3.2 Egg Production and Growth Performance

A significant drop in average daily feed intake (ADFI) and an improved feed conversion ratio (FCR) were found as a result of the feeding with MAR diets when compared with NC diet (*p* < 0.05; Table 3). However, total numbers of eggs, egg weight and egg mass were not significantly different in both groups.

**TABLE 3:** Egg production, average daily feed intake (ADFI) and feed conversion ratio (FCR) in laying hens fed NC and margarine (MAR) diets over a 10‐week period.

Parameters	NC group	MAR group	*p* value
Average egg weight	58.89 ± 2.65	60.58 ± 4.80	0.10
Egg number	92.77 ± 16.16	91.50 ± 11.42	0.33
ADFI	131.72^a^ ± 59.24	97.53^b^ ± 6.21	0.001
Laying rate	0.69 ± 0.12	0.68 ± 0.08	0.34
Egg mass	41.06 ± 7.55	41.45 ± 4.25	0.27
FCR	3.29^a^ ± 1.65	2.37^b^ ± 0.27	0.05

*Note*: FCR, ADFI, egg weight, egg number, egg mass and laying rate in laying hens. (A) FCR was calculated by dividing egg mass ADFI/egg mass; (B) ADFI was calculated by a number of chickens × 1000/feed consumption. (C) Egg weight was calculated daily and averaged. (D) Egg numbers were recorded daily for individual hens in the group. (E) Egg mass was calculated as number of chicken/egg production. (F) Laying rate calculated as number of chicken/egg number. Means bearing different superscript letters differ significantly *p* < 0.05.

### 3.3 Histological Features

**FIGURE 1:**
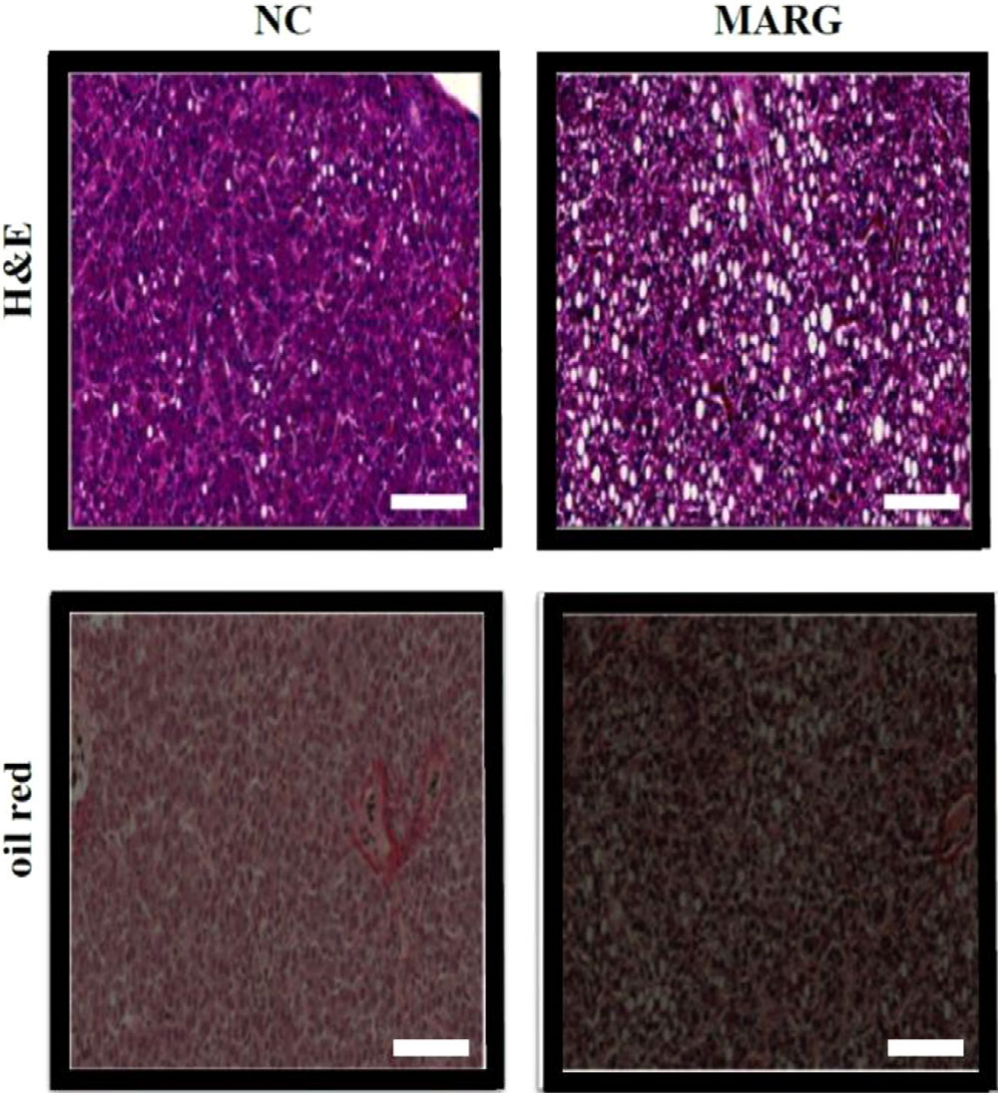
Representative histological liver sections of laying hens fed NC and MAR diets. Sample histological images of liver sections stained with haematoxylin and eosin (H&E) (top row) as well as oil red O (bottom row) are displayed. The liver specimens were collected after 62 weeks of the experiment and show steatosis, ballooning, lobular and portal inflammation cells; images were taken using a magnification of 200‐fold. MAR, margarine; NC, natural milk cream.

Histological examination indicated a higher prevalence of advanced steatosis (stages 3–4) and more frequent inflammatory foci in MAR‐fed hens compared with controls. Although these differences were not statistically significant, fibrosis severity showed a trend toward a higher proportion of advanced cases in the MAR group (*p* = 0.073) (Figure 1, Table 4).

**TABLE 4:** Histological scoring of liver tissue in laying hens fed NC and margarine (MAR) diets.

Histology feature	Ranking	NC (%)	MARG (%)	Median rank (NC vs. MARG)	Mann–Whitney U (*p* value)
**Steatosis**	>5%	5 (55.6)	3 (33.3)	NC = 0.0, MARG = 1.0	*U* = 29.5, *p* = 0.327
	5%–30%	2 (22.2)	2 (22.2)		
	33%–66%	1 (11.1)	2 (22.2)		
	>66%	1 (11.1)	2 (22.2)		
**Lobular inflammation**	No foci	6 (66.7)	5 (55.6)	NC = 0.0, MARG = 0.0	*U* = 33.0, *p* = 0.480
	<2 foci	2 (22.2)	1 (11.1)		
	2–4 foci	1 (11.1)	2 (22.2)		
	>4 foci	0 (0)	1 (11.1)		
**Portal inflammation**	None	7 (77.8)	5 (55.6)	NC = 0.0, MARG = 0.0	*U* = 31.5, *p* = 0.369
	Mild	1 (11.1)	2 (22.2)		
	>Mild	1 (11.1)	2 (22.2)		
**Liver cell injury**	None	7 (77.8)	5 (55.6)	NC = 0.0, MARG = 0.0	*U* = 37.5, *p* = 0.506
	Few	2 (22.2)	2 (22.2)		
	Many	1 (11.1)	2 (22.2)		
**Fibrosis stage**	None	5 (55.6)	2 (22.2)	NC = 0.0, MARG = 2.0	*U* = 20.5, *p* = 0.073
	Stage 1	2 (22.2)	1 (11.1)		
	Stage 2	1 (11.1)	2 (22.2)		
	Stage 3	1 (11.1)	3 (33.3)		
	Stage 4	0 (0)	1 (11.1)		

*Note*: Histological evaluation of liver sections. Parameters include steatosis, lobular inflammation, hepatocyte ballooning and fibrosis. Scoring was based on the NASH Clinical Research Network criteria. Values are presented as mean ± SD (*n* = 9). Statistical significance was determined using the Kruskal–Wallis test followed by pairwise Mann–Whitney *U*‐test.

### 3.4 Biochemical Indices

**FIGURE 2:**
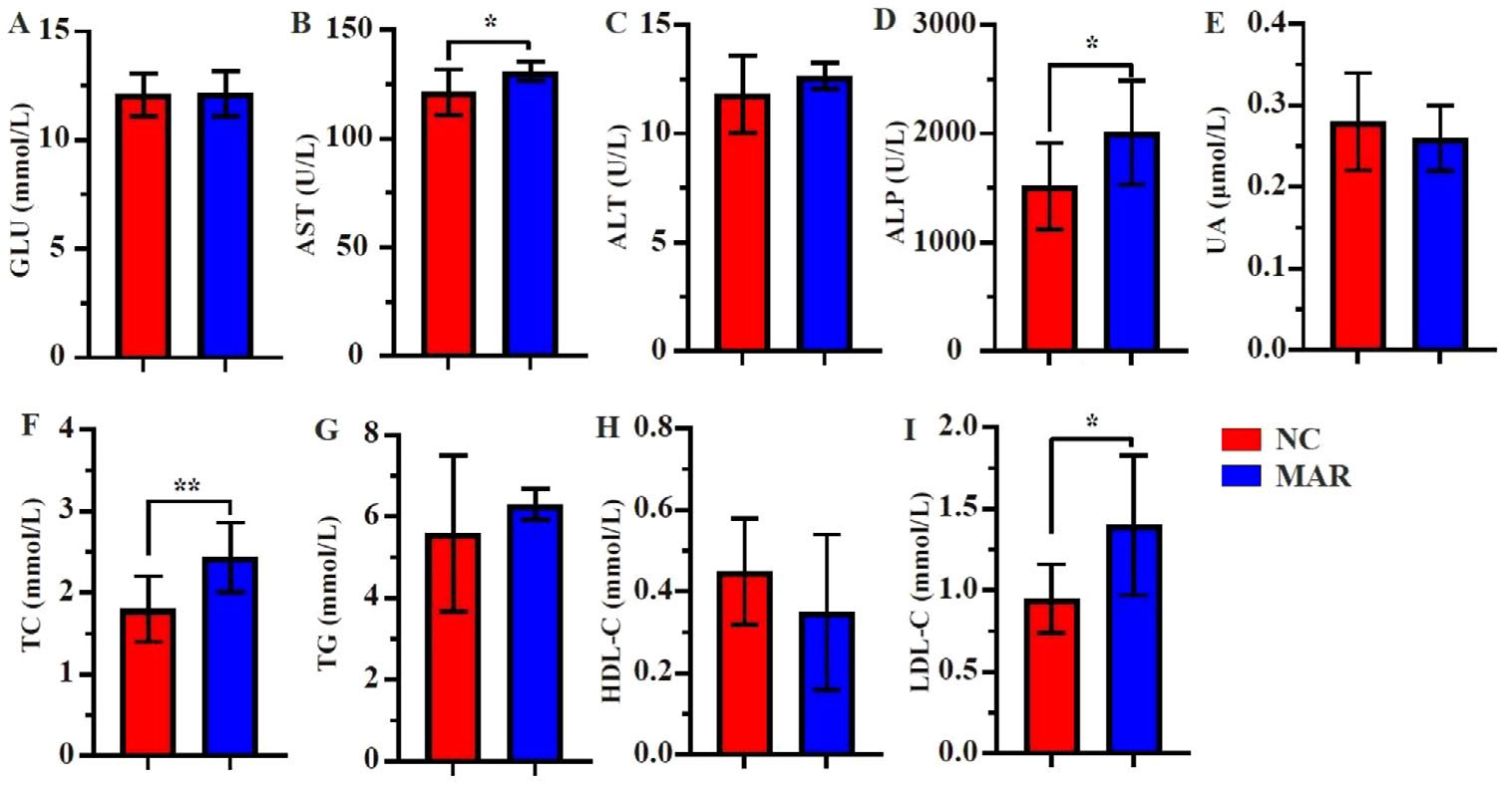
Serum biochemical indices of liver function and lipid metabolism in laying hens fed NC and MAR diets. Blood biochemical parameter in serum: (A) Glu, glucose; (B) AST, aspartate aminotransferase; (C) ALT, alanine aminotransferase; (D) ALP, alkaline phosphatase; (E) UA, urea; (F) TC, total cholesterol; (G) TG, triglyceride; (H) HDL‐C, high‐density lipoprotein; (I) LDL‐C, low‐density lipoprotein. Significance levels: *p < 0.05. MAR, margarine; NC, natural milk cream.

**FIGURE 3:**
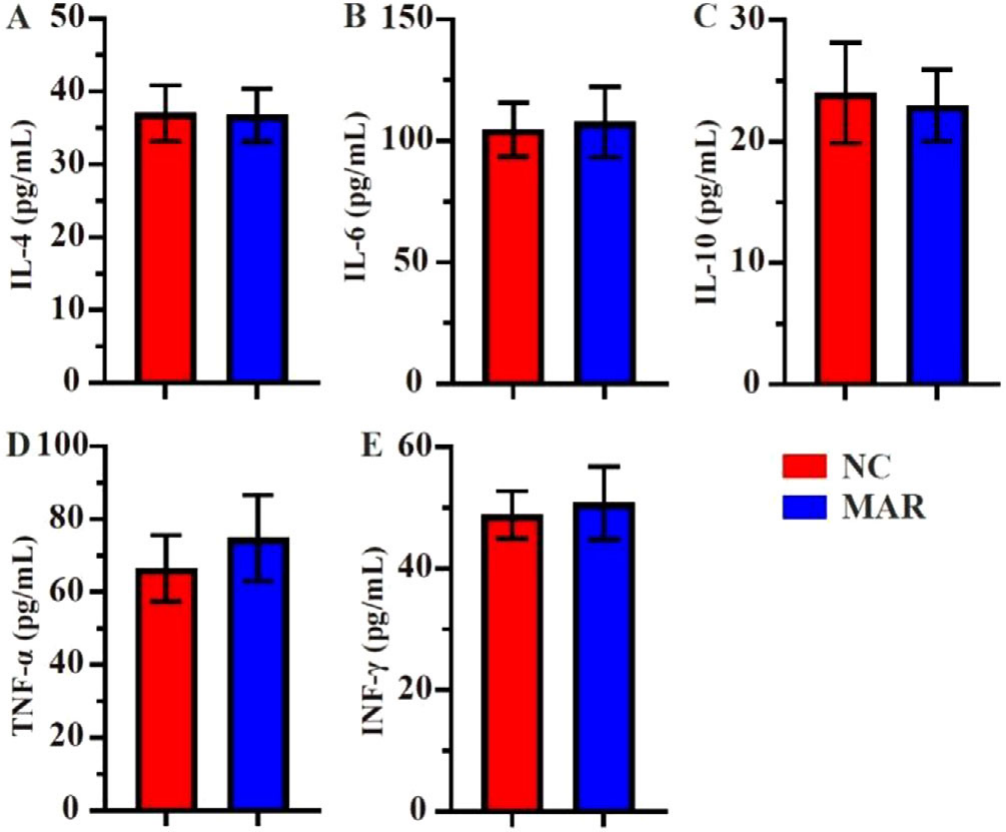
Biochemical indices of hens in laying hens fed NC and MAR diets. Blood biochemical parameter in serum. (A) IL‐4, interleukin 4; (B) IL‐6, interleukin 6; (C) IL‐10, interleukin 10; (D) TNF‐α, tumour necrosis factor; (E) IFN‐γ, interferon gamma. Significance levels: *p < 0.05. MAR, margarine; NC, natural milk cream.

**FIGURE 4:**
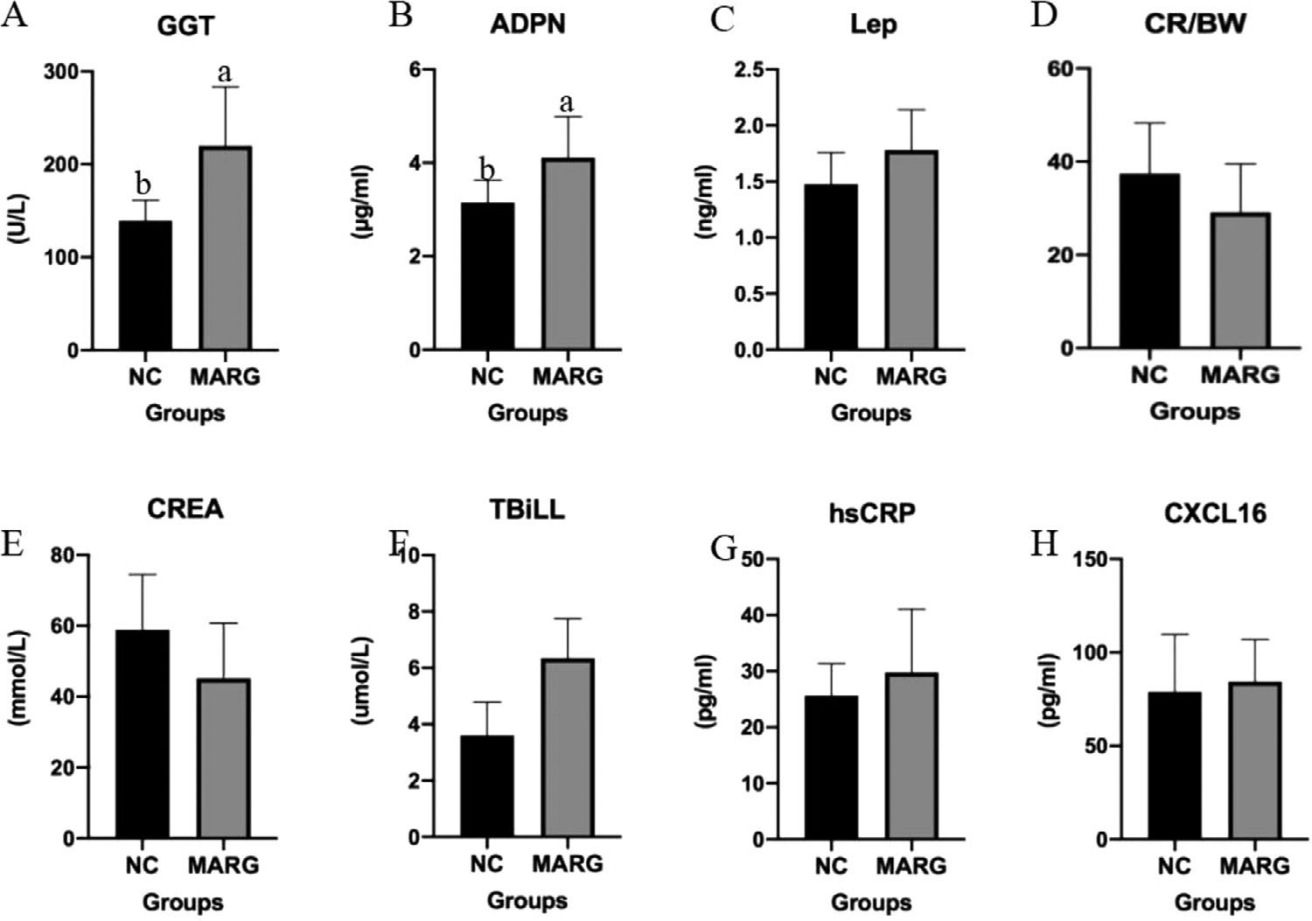
Biochemical indices of hens fed NC and MAR diets: (A) GGT, gamma‐glutamyl transferase; (B) ADPN, adiponectin; (C) Lep, leptin; (D) CR/BW, creatinine/body weight; (E) CREA, creatinine; (F) TBiLL, total bilirubin; (G) hsCRP, high‐sensitivity C‐reactive protein; (H) CXCL16, chemokine (C–X–C motif) ligand 16. Significance levels: *p < 0.05. MAR, margarine; NC, natural milk cream.

MAR‐fed hens showed higher serum ALP, AST, TC and LDL‐C levels as compared with NC group (*p* < 0.05, Figure 2), whereas no significant differences were found in ALT and HDL‐C levels. The study result demonstrates that between two groups, pro‐inflammatory cytokines (IFN‐γ, TNF‐α, IL‐6) and anti‐inflammatory adipokines (IL‐10 and IL‐4) were not significantly different (Figure 3). GGT and adiponectin levels were higher in the MAR group (*p* < 0.05), though leptin and creatinine remained unaffected (Figure 4).

### 3.5 Alteration of Gut Microbiota

**FIGURE 5:**
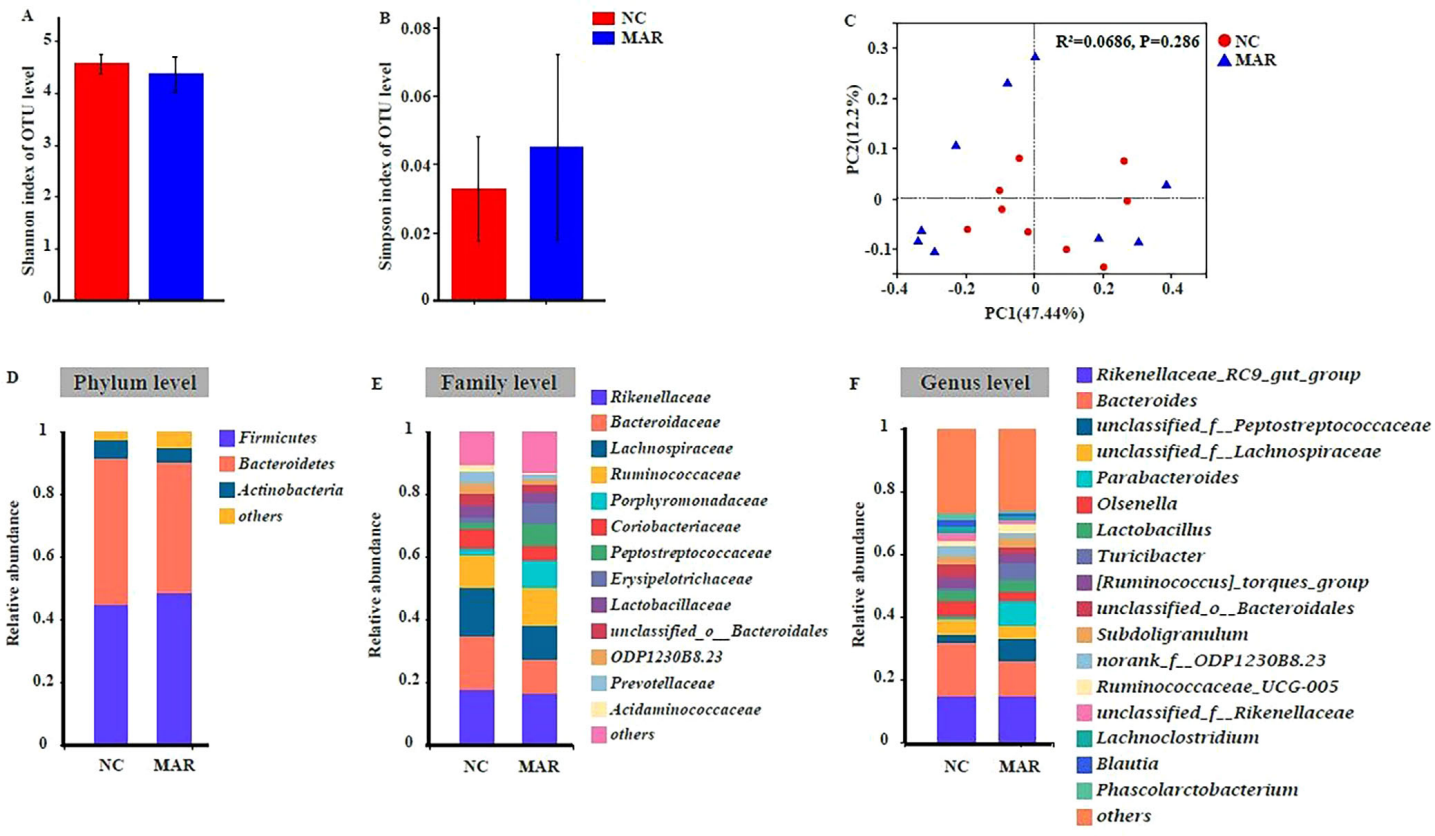
Effects of NC and MAR diets on caecal microbiota in laying hens. (A) Shannon and (B) Simpson alpha‐diversity indices at the OTU level. (C) Principal coordinates analysis (PCoA, Bray–Curtis distance) of microbial community structure between NC and MAR groups. Each point represents one sample; closer points indicate more similar species composition. (D) Relative abundance of caecal microbiota at the phylum level, (E) family level and (F) genus level. NC group, high‐energy diet with natural milk cream; MAR group, high‐energy diet with trans‐fat margarine.

**FIGURE 6:**
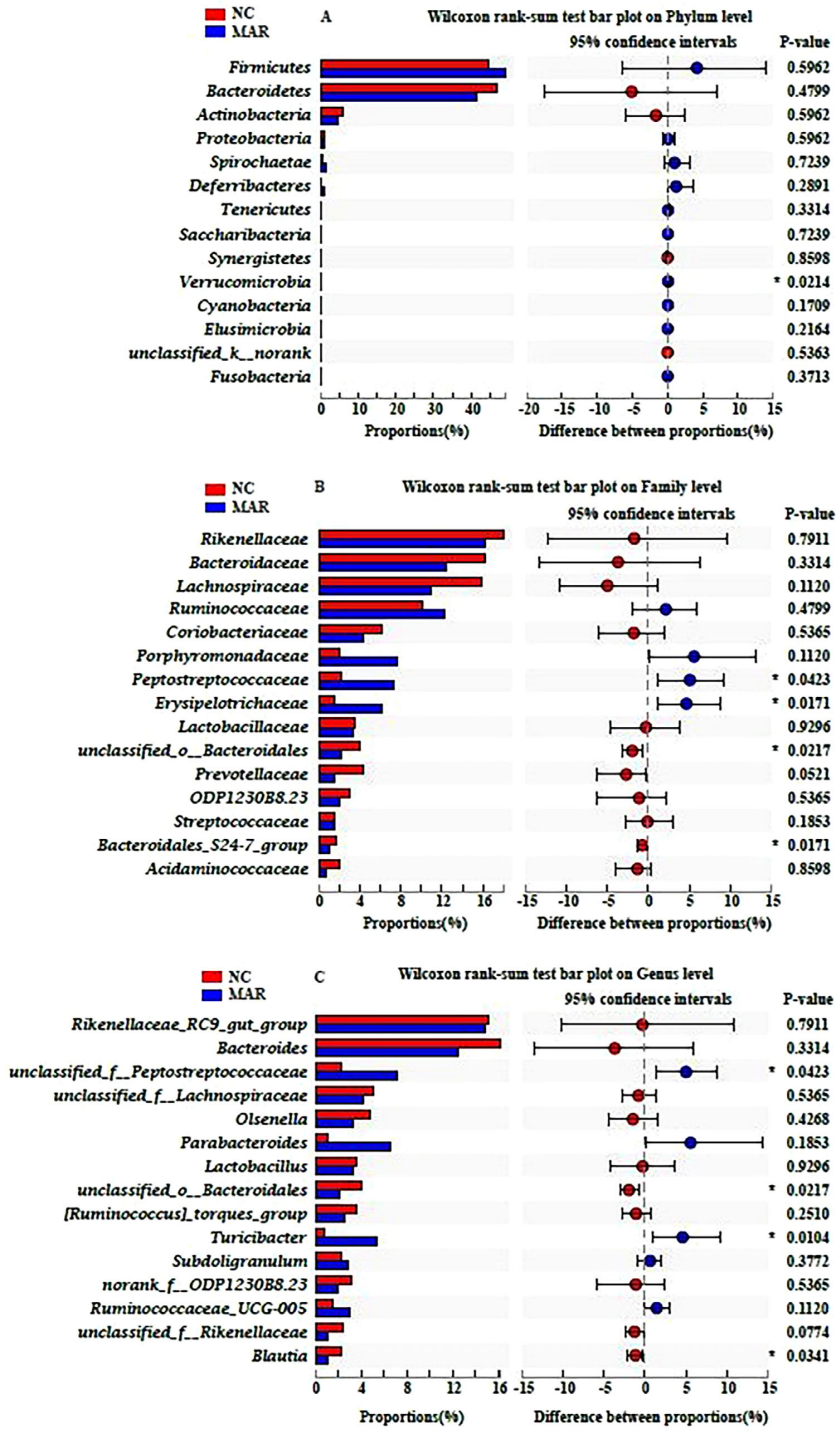
Wilcoxon rank‐sum test of caecal microbiota: (A) phylum, (B) family and (C) genus levels. Bacterial clades that differed significantly between NC and MAR groups are shown (*p < 0.05). MAR, margarine; NC, natural milk cream.

The results of microbial richness showed that Shannon and Simpson indices were lower in MAR group as compared to NC group (Figure 5A,B). The principal coordinate analysis (PC) was calculated between the two groups and showed differences in microbial communities in NC and MAR groups (Figure 5C). Firmicutes and Bacteroidetes were the predominant phyla in both groups, with MAR showing a higher but non‐significant Firmicutes to Bacteroidetes ratio (Figure 5D). Both groups were dominated by four families: Rikenellaceae, Bacteroidaceae, Lachnospiraceae and Ruminococcaceae (Figure 5E), whereas at the genus level, Rikenellaceae RC9 gut‐group, Bacteroides and unclassified Peptostreptococcaceae were dominant (Figure 5F). The abundance of the Verrucomicrobia phylum was elevated in MAR group (0.14%, *p* < 0.05, Figure 6A). whereas the Peptostreptococcaceae family was higher, with the genus unclassified Peptostreptococcus; however, unclassified Bacteroidales and Turicibacter were also elevated in MAR group (*p* < 0.05, Figure 6B,C). Interestingly, the *Blautia* genus was reduced.

## 4 Discussion

The objective of this research was to evaluate the effect of two high‐fat diets, formulated with similar energy levels but differing in fatty acid composition, on laying hens over a 10‐week period. A limitation of this study is the lack of precise energy matching between the two diets. Although they were designed to be energy‐comparable, differences in fatty acid composition and resulting digestibility likely led to unequal AME intake. MAR, though devoid of trans fats, is rich in lauric and stearic acids, which have lower digestibility and bioavailability compared to the palmitic and oleic acids dominant in milk cream. These energy‐related and compositional differences may have contributed to the increased liver fat accumulation and metabolic disturbances observed in the MAR group, emphasizing the importance of fat quality—not just total energy—in influencing liver health and lipid metabolism. The results suggest that dietary fats with differing fatty acid compositions exert substantial effects on lipid metabolism, egg production, liver health and gut microbiota composition. Compared to the NC group, MAR‐fed hens showed higher serum levels of total cholesterol and LDL‐C, along with increased liver and abdominal fat deposition. Previous research has demonstrated that both the source of dietary fat and the specific fatty acid profile significantly influence hepatic lipid metabolism and activate different metabolic pathways (Hodson et al. 2020). These findings support that the MAR diet—characterized by a high content of lauric and stearic acids—played an important role in promoting hepatic lipid accumulation. Lauric acid, despite being a medium‐chain fatty acid, has been shown to elevate LDL‐C levels (Schwingshackl and Schlesinger 2023) and may exert adverse metabolic effects (Eyres et al. 2016; Sacks et al. 2017), including promotion of atherogenic profiles and increased hepatic fat storage (Neelakantan et al. 2020). Stiuso et al. (2014) also suggested that stearic acid may contribute to hepatic fat accumulation, which aligns with the outcomes observed in the MAR group of this study. Although the lipotoxic properties of palmitic acid have been associated with increased hepatic fat deposition and insulin resistance (Murru et al. 2022), our findings indicate that hens fed the NC diet—rich in both palmitic and oleic acids—had lower liver fat accumulation than those fed the MAR diet. This protective effect may result from the synergistic interaction of palmitic and oleic acids. Oleic acid, a monounsaturated fatty acid, has been shown to reduce liver fat accumulation (Castillo et al. 2024) and mitigate the harmful effects of palmitic acid on hepatic tissue (Zeng et al. 2020). These interactions are consistent with the histological and biochemical results observed in our study. The study revealed that MAR‐fed hens had higher liver weight and fat accumulation. This could result from the fatty acid composition in the diet, particularly lauric acid, which may induce hepatic lipogenesis and increase the risk of developing fatty liver disease, as supported by Saraswathi et al. (2020). Significantly elevated levels of AST, ALP and GGT were observed in the MAR‐fed hens, suggesting liver stress and possible dysfunction. Higher GGT levels may indicate bile duct damage or oxidative stress, both of which are key markers of NAFLD and are commonly associated with high saturated fat intake or diets low in antioxidant capacity (Khonyoung et al. 2023). Similar metabolic disturbances linked to saturated fats and inadequate antioxidant defence have also been reported in broilers under heat stress conditions (Ouyang et al. 2022) and in studies using essential oils to mitigate oxidative and inflammatory responses (Moharreri et al. 2021). Evidence also suggests that lauric acid may promote obesity and hepatic lipid accumulation (Buettner et al. 2013) and has been associated with elevated plasma triglyceride and free fatty acid concentrations in mouse models (Jung et al. 2011). Our study further revealed that hens fed the MAR diet had lower levels of lipoprotein lipase (LPL). As LPL plays an important role in the hydrolysis of triglycerides in lipoproteins and facilitates the uptake of free fatty acids by tissues, reduced LPL activity could contribute to fat accumulation in the liver and increase risk factors associated with cardiovascular disease and metabolic syndrome (Judd et al. 1998). The results from gut microbiota analysis revealed that MAR‐fed hens exhibited lower microbial richness and distinct alterations in specific bacterial populations. These findings align with previous studies on the impact of high‐fat diets on gut health and microbiome structure (Machate et al. 2020), as well as trials using microencapsulated essential oils to restore microbial balance under enteric stress (Moharreri et al. 2021). A decline in microbial diversity, as evidenced by decreased Shannon and Simpson index values. Although not statistically significant, such reductions are typically associated with adverse health effects, including metabolic disorders and inflammatory diseases. Lower microbial diversity also reflects a less resilient gut microbiome, making it more vulnerable to dysbiosis and pathogen overgrowth (Patterson et al. 2014). The slight, but not statistically significant, increase in the relative proportion of Firmicutes to Bacteroidetes detected in the MAR group reflects findings from other studies that associate this ratio with obesity and metabolic diseases (Hamid et al. 2019). High‐fat diets—particularly those rich in saturated fats—have been shown to alter the gut microbiota by favouring the growth of Firmicutes over Bacteroidetes. This microbial shift has been linked to increased energy harvest from the diet and enhanced fat deposition, thereby raising the risk of metabolic dysregulation in poultry. In our study, we observed an increase in the phyla Verrucomicrobia and the family Peptostreptococcaceae. At the genus level, increases were seen in unclassified Peptostreptococcus, unclassified Bacteroidales and Turicibacter, suggesting the MAR diet. Although not statistically significant, these shifts suggest that the MAR diet may influence gut microbial composition. Recent studies have associated Verrucomicrobia abundance with liver disease and metabolic syndromes, implicating it in the progression of hepatic dysfunction (Perlin et al. 2024). Additionally, the rise in the Peptostreptococcaceae family and related genera has been associated with cognitive dysfunction and severe metabolic impairment in the context of liver disease (Efremova et al. 2024). A relative decline in *Blautia* was observed in the MAR group. While not statistically significant, this reduction could contribute to an unfavourable microbial and metabolic environment, consistent with previous studies. *Blautia* contributes to microbial homeostasis, anti‐inflammatory activity and intestinal barrier integrity. Its decline could create a less favourable metabolic environment, increase liver pathology and disrupt lipid regulation, as supported by recent findings (Chen et al. 2021; Li et al. 2020). This reduction may also impair host resistance to pathogenic colonization and inflammation (Al‐Khalaifah et al. 2022). Overall, the descriptive shifts in gut microbiota suggest that the MAR diet may influence liver health and lipid metabolism not only directly but also indirectly through changes in gut microbial ecology. These findings highlight the importance of fat quality, rather than inclusion level alone, though most observed differences were not statistically significant. In summary, findings from this work may support new dietary strategies for the prevention or mitigation of fatty liver disease in poultry, with potential translational relevance to human health, by advancing our understanding of fat–microbiota–liver interactions. Nevertheless, a limitation of this study is the absence of egg quality data, which restricts the direct applicability of results to poultry production systems where egg performance and quality are critical outcomes. Future studies integrating egg quality parameters with metabolic and microbial assessments will provide a more comprehensive picture. From a practical perspective, these results highlight the importance of selecting dietary fat sources carefully in layer feed formulation, as improving fat quality may help reduce the incidence of fatty liver haemorrhagic syndrome, enhance hen welfare and ultimately support more sustainable egg production.

## Conclusions

This study demonstrated that an MAR‐based diet, rich in lauric and stearic acids, elevated serum markers of liver damage (AST, ALP, GGT) and disrupted gut microbial diversity in laying hens, compared to a milk cream‐based diet. These findings underscore the importance of dietary fat quality in shaping hepatic health and microbiota composition. The MAR diet also led to reduced LPL levels and a decline in beneficial bacteria such as *Blautia*, suggesting possible links to metabolic and microbial dysregulation. These outcomes provide new insights into the gut–liver axis and highlight the potential risks of saturated fat‐rich diets in poultry. Further studies are recommended to explore mitigation strategies and validate the role of gut microbiota as a therapeutic target in fatty liver disease.

## Author Contributions

Conceptualization: H.H. and Q.G.M. Methodology: H.H., S.M.H., L.H.Z., and Q.G.M. Investigation: H.H., Y.J.L., and W.X.L. Formal Analysis and Data Curation: H.H., L.J.X., and S.M.H. Writing—Original Draft Preparation: H.H. Writing—Review and Editing: L.J.X., M.R.Q., and Q.G.M. Visualization and Supervision: H.H. and S.M.H. Project Administration and Funding Acquisition: L.J.X. and Q.G.M. All authors have read and agreed to the published version of the manuscript.

## Disclosure

The funders had no role in the design of the study; in the collection, analyses, or interpretation of data; in the writing of the manuscript; or in the decision to publish the results.

## Ethics Statement

The study was carried out in compliance with the ARRIVE guidelines. The animal trial was approved by the Animal Ethics Committee at University. There were no vulnerable populations involved, and no endangered species were used in the experiments. Farm managers gave permission for their animal samples to be used in this study. Experimental animals in Beijing, China, were treated humanely at all times and in accordance with the Ministry of Science and Technology's Guide for Experimental Animal.

## Consent

The authors have nothing to report.

## Conflicts of Interest

The authors declare no conflicts of interest.

## Peer Review

The peer review history for this article is available at https://www.webofscience.com/api/gateway/wos/peer‐review/10.1002/vms3.70665.

## Data Availability

All data generated or analysed during this study are included in the article. Any additional information regarding the data can be obtained from the corresponding author upon reasonable request.
